# One gene to rule them all – clinical perspectives of a potent suppressor of cytokine signaling – SOCS1

**DOI:** 10.3389/fimmu.2024.1385190

**Published:** 2024-04-22

**Authors:** Julia Körholz, Lan-Sun Chen, Timmy Strauss, Catharina Schuetz, Alexander H. Dalpke

**Affiliations:** ^1^ Department of Pediatrics, Faculty of Medicine and University Hospital Carl Gustav Carus, Technische Universität Dresden, Dresden, Germany; ^2^ University Center for Chronic Immunodeficiencies (UCID), Faculty of Medicine and University Hospital Carl Gustav Carus, Technische Universität Dresden, Dresden, Germany; ^3^ Department of Infectious Diseases, Medical Microbiology and Hygiene, Medical Faculty, University Heidelberg, Heidelberg, Germany; ^4^ University Hospital Heidelberg, Heidelberg, Germany; ^5^ University Center for Rare Diseases, Faculty of Medicine and University Hospital Carl Gustav Carus, Technische Universität Dresden, Dresden, Germany

**Keywords:** SOCS1, SOCS1-HI, JAKinhibitors, SOCS1-immunity, multisystem immune dysregulation, monogenic hyperinflammation

## Abstract

The discovery of Suppressor of Cytokine Signaling 1 (SOCS1) in 1997 marked a significant milestone in understanding the regulation of Janus kinase/Signal transducer and activator of transcription (JAK/STAT) signaling pathways. Subsequent research deciphered its cellular functions, and recent insights into SOCS1 deficiencies in humans underscored its critical role in immune regulation. In humans, SOCS-haploinsufficiency (SOCS1-HI) presents a diverse clinical spectrum, encompassing autoimmune diseases, infection susceptibility, and cancer. Variability in disease manifestation, even within families sharing the same genetic variant, raises questions about clinical penetrance and the need for individualized treatments. Current therapeutic strategies include JAK inhibition, with promising results in controlling inflammation in SOCS1-HI patients. Hematopoietic stem cell transplantation and gene therapy emerge as promising avenues for curative treatments. The evolving landscape of SOCS1 research, emphasizes the need for a nuanced understanding of genetic variants and their functional consequences.

## 1 Introduction

The year 1997 marked the beginning of the SOCS1 story. Despite significant knowledge about Janus kinase/Signal transducer and activator of transcription (JAK/STAT) signaling pathways, the regulation of these pathways remained rather unexplored. Excitingly, three distinct research groups embarked on a journey that uncovered a novel protein capable of regulating intracellular JAK/STAT signaling. Whereas knowledge of the cellular function of SOCS1 has been explored extensively since then, it is only recently that the first patients with SOCS1 deficiencies have been described. We now summarize our clinical perspective on SOCS1 in this article.

## 2 SOCS1 perspectives

### 2.1 How it all began

The Australian research group led by Hilton and colleagues, set out to identify cDNAs encoding proteins with the ability to suppress cytokine signaling. Murine monocytic leukemic M1 cells, able to differentiate into mature macrophages were infected with cDNAs and explored for unresponsiveness to IL-6 stimulation. They isolated a small cDNA-insert through PCR and named it SOCS1. High expression of SOCS1 was detected, especially in thymic, spleen, and lung tissues pointing towards a function in regulating immune responses. Structural analyses revealed the presence of Src-homology region 2 (SH2) and SOCSbox domains, shared features with other proteins in what would later be termed the SOCS family. *In vitro* experiments demonstrated a specific effect of SOCS1 on cytokine signal transduction within the JAK/STAT-signaling pathway (1).

Simultaneously, the Tokyo-based research group led by Yoshimura, who had previously discovered the CIS protein with binding potential to the IL-3 receptor, utilized a yeast two-hybrid cDNA library to screen for proteins binding to JAK. This led to the identification of JAB (JAK-binding protein). *In vitro* experiments confirmed binding of JAB to JAK2, resulting in reduced tyrosine phosphorylation and negative regulation of JAK/STAT signaling. They noted that the SH2 domain alone was insufficient for the kinase inhibitory activity of JAB (2).

The third group, Kishimoto and colleagues from Osaka, developed a monoclonal antibody targeting a sequence motif in the SH2 domain of STAT3. Using this antibody, they screened a murine thymus cDNA library and identified 20 unknown genes, including a novel gene they named SSI-1 (STAT-induced STAT inhibitor-1). SSI-1 expression was prominent in murine spleen, lungs, and testis. Like the other groups, Kishimoto et al. observed an inhibitory effect of SSI-1 on the JAK/STAT signaling pathway (3).

These groundbreaking discoveries culminated in back-to-back publications in *Nature* (1–3).

### 2.2 Structure and activity of SOCS1

**Figure 1:**
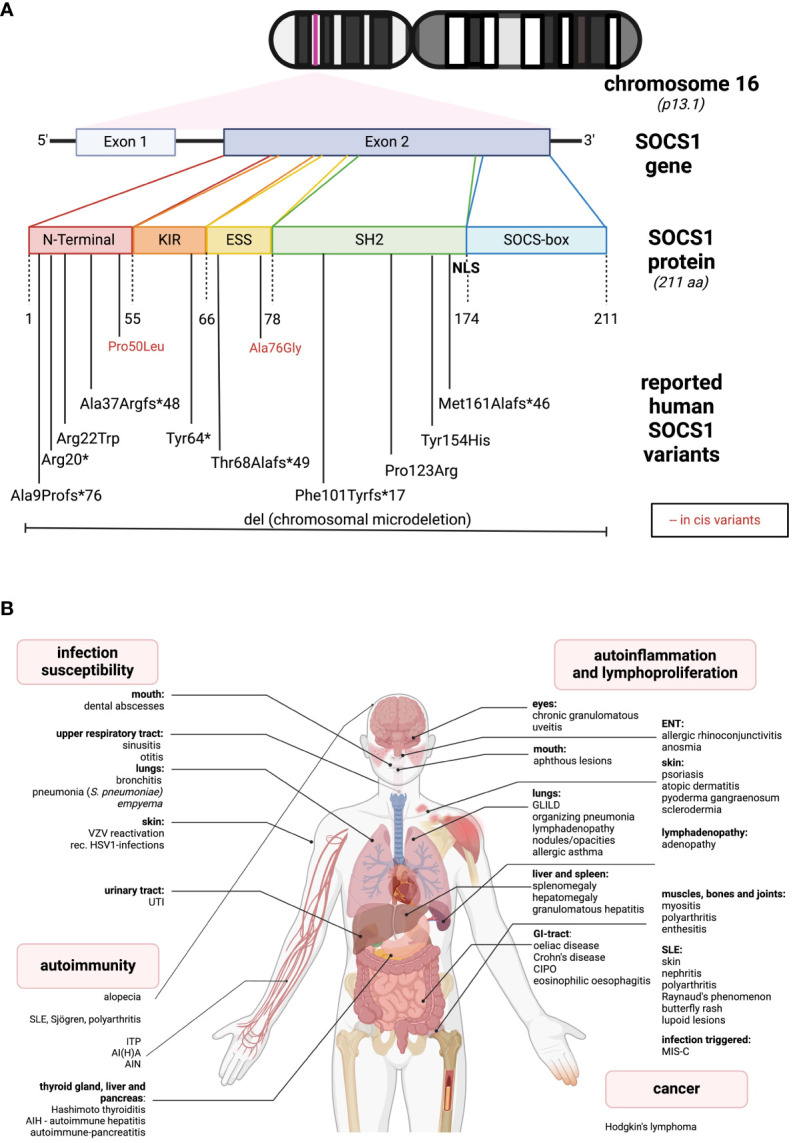
Genetic and clinical spectrum of SOCS1-haploinsufficiency **(A)** The *SOCS1* gene is located on chromosome 16. It consists of two exons while only one is coding. The protein comprises different sub-domains: KIR, kinase inhibitory region; ESS, extended SH2-subdomain; SH2, Src-homology 2 domain; NLS, nuclear localization sequence. In the lower part, all the so far reported SOCS1 variants leading to SOCS1-HI are listed according to their location. Red letters indicate two reported in cis variants, in combination leading to a disease phenotype. **(B)** So far reported clinical spectrum of SOCS1-HI in humans distinguished between infection susceptibility, autoimmunity, autoinflammation and cancer. The figures were created using biorender.com.

SOCS1, located on chromosome 16, is a relatively compact gene composed of two exons, with one coding exon only. The gene encodes the 211-amino acid (AA) SOCS1 protein (**Figure 1A**). A distinctive feature of SOCS1 and SOCS3 within the SOCS family is, in addition to the shared SH2- and the SOCSbox domains, the presence of an N-terminal Kinase Inhibitory Region (KIR), which acts as a pseudosubstrate disrupting JAK tyrosine kinase activity (4). The SH2-domain of SOCS1 directly binds to the activation loop of various JAKs, inhibiting JAK activation and subsequent STAT phosphorylation (4). Both the SH2 and KIR domains are crucial for SOCS1’s cytokine suppressor function *in vivo* (5). The C-terminal SOCS box domain interacts with elongin B/C, Cullins, and the RING-finger domain-only protein RBX2, forming an E3 ubiquitin ligase. This complex mediates proteasomal degradation of associated signaling intermediates (4). In 2008, Baetz and colleagues identified a nuclear localization signal (NLS) in SOCS1 between the SH2 and SOCS box domains (6). This intranuclear action allows SOCS1 to regulate additional signaling pathways, such as NFκB, by inducing proteasomal degradation (7).

Whereas SOCS1 expression in homeostasis is low in most cells, a rapid induction can be observed upon stimulation by type I and II cytokine receptors. SOCS1 thus acts as a classical temporal feedback inhibitor. Induction and activity are promiscuous for different JAK/STAT members: SOCS1 interacts directly with JAK1, JAK2, and TYK2 primarily through the pseudosubstrate KIR. Additionally, it can bind to phosphotyrosine residues on cytokine receptors such as IL-2Rβ via its SH2 domain. Consequently, SOCS1 predominantly limits the actions of STAT1 but also affects STAT4, STAT5, and STAT6 (8). Insights from knockouts argue for a peculiar significance for IFN signaling, most importantly IFN-γ.

### 2.3 SOCS1 functional activity: regulating immunity and beyond

The importance of SOCS1 in immune cell differentiation and regulation is most evident in T cells, influencing CD4/CD8 ratios, thymic T-cell development, and T-helper cell polarization. In SOCS1 deficient mice, aberrant CD4/CD8-ratios have been observed further indicating a role of SOCS1 in thymic T-cell development (4). SOCS1 deficiency results in a Th1-dominant phenotype with suppressed Th17 cells, likely due to IFN-γ hyperproduction or overstimulation (4). Additionally, SOCS1 is crucial for regulatory T cell (Treg) function, as SOCS1-deficient Tregs exhibit reduced Foxp3 expression (4). Furthermore, SOCS1 plays a role in dendritic cell (DC) maturation and activation, with SOCS1-deficient DCs showing hyperactivation in response to cytokine stimulation (9).

SOCS1’s influence extends beyond the hematopoietic system, as it is highly expressed in various tissues (10, 11). This diverse expression pattern might contribute to clinical phenotypes of SOCS1 insufficiency and must be kept in mind when discussing curative treatment options such as hematopoietic stem cell transplantation (HSCT). The involvement of SOCS1 in different signaling pathways has distinct effects on immune dysregulation and local immunity. For example, the nuclear localization of SOCS1 seems to be crucial for local immunity in the lung, as mice lacking the NLS of SOCS1 develop low-grade pulmonary inflammation (12).

### 2.4 SOCS1 and cancer

#### 2.4.1 SOCS1 in inflammation-associated cancer development

SOCS1’s interaction with various intracytosolic and intranuclear proteins also plays a role in cancer development, with altered expression observed in various tumor entities. Moreover, SOCS1 plays a pivotal role in controlling inflammation, a critical factor in cancer development. Different alterations of SOCS1 expression contribute to this mechanism, beginning with SOCS1 silencing through hypermethylation of its promoter region, observed in diverse malignant diseases such as pancreatic carcinoma, acute myeloid leukemia, and hepatocellular carcinoma (HCC) (13). In human HCC cell cultures with SOCS1 inactivation by hypermethylation of the promotor region, Yoshikawa and colleagues observed reduced growth rates when SOCS1 function was restored, indicating the importance of the constitutive activation of the JAK/STAT pathway in the development of HCC (14). In other malignancies, including breast cancer and multiple myeloma, elevated expression of microRNAs targeting the *SOCS1* gene, emerges as a mechanism contributing to cancer development (13).

Additionally, SOCS1 restricts the capacity of interferons (IFNs) to enhance tumor immunity (13). For instance, SOCS1 deficiency enhances the antitumor effects of IFN-α *in vivo*, as demonstrated in a malignant melanoma mouse model. SOCS1^-/-^, IFN-γ^-/-^ mice showed a more significant response to IFN-α treatments, leading to better cure rates compared to SOCS1 wildtypes (15). SOCS1 can further limit anti-proliferative and pro-apoptotic effects of IFNs: when downregulating *SOCS1* gene expression in colon- and melanoma- cancer cell lines, IFN-γ treatment led to reduced proliferation and induced interferon-sensitive response element (ISRE)-mediated transcriptional activity (16).

#### 2.4.2 SOCS1 as direct tumor suppressor and oncogene

Beyond its interactions with key proteins in cytokine signaling pathways, SOCS1 engages directly with tumor suppressors and oncogenes, intricately regulating their expression.

In the nucleus, the SOCS1-SH2 domain interacts with the N-terminal transactivation domain of p53, enhancing p53-phosphorylation and subsequent DNA-binding, thereby amplifying transcriptional activity. Simultaneously, the SOCSbox domain interacts with ATM/ATR, further modulating p53 function. SOCS1 facilitates proteasomal degradation of p21, which acts as oncogene when retained in the cytosolic compartment. SOCS1-mediated proteasomal degradation also limits various signaling pathways involving the growth factor receptor tyrosine kinase (RTK), a critical mechanism in tumor growth, e.g. in HCC (13).

However, SOCS1 has a dichotomous role in cancer development: It can also function as an oncogene, as evidenced by its overexpression at protein or mRNA levels in solid tumors like melanoma, directly correlating with heightened tumor invasion and growth (13, 17). When SOCS1 was silenced in Mel526 melanoma cells, proliferation was reduced (18). SOCS1 has recently been identified as direct transcriptional target of Hedgehog (Hh/GLI) signaling in human medulloblastoma cell lines, whereas silencing of SOCS1 promoted reduced medulloblastoma growth *in vitro* (19).

The question arises, why SOCS1 can display both tumor suppressing and promoting activity within different and sometimes the same cell type (13). As *SOCS1* itself is not very prone to mutations, different SOCS1 expression levels may help explain these opposed actions. SOCS1 availability may be regulated by phosphorylation in different AA positions influencing its contribution to JAK/STAT and proteasomal degradation pathways, as well as its interaction with other oncogenes such as BCR-ABL (13).

In sum, the experimental observations above illustrate the complex interplay between pro- and anti-oncogenic pathways at the molecular and cellular levels, and how tumor immunology is intertwined with dysregulated inflammatory pathways.

### 2.5 SOCS1 and virus infections

Known for its role in suppressing intracellular JAK/STAT signaling, SOCS1 emerged as a key player in regulating viral immune evasion. The intriguing twist comes as viruses exploit SOCS proteins, turning them into virulence factors to subvert antiviral immune responses. Evidence has shown that the overexpression of SOCS1 enhances virus replication in HCV infections by negating the antiviral effects of Type I IFNs (20). Similarly, for SARS-CoV-2, the accessory viral protein ORF3a induces intracellular SOCS1 expression and proteasomal degradation of signaling intermediates in the JAK/STAT pathway, such as JAK2 (21). This process results in a reduction of antiviral immune responses, highlighting SOCS1 as a potential therapeutic target for virus infections.

SOCS1/3 antagonists have shown promise in treating viral infections, including HSV-1, Influenza A, and vaccinia virus infections, both *in vitro* and in mice (22, 23). These antagonists are also under discussion as potential therapeutic targets for severe SARS-CoV-2 infections (23).

### 2.6 SOCS1 in animal models

Altered SOCS1 function has significant implications for immune function *in vivo*. Mice with total SOCS1 deficiency (SOCS1^-/-^) have a normal phenotype at birth compared to SOCS1^+^/^+^ wildtype and SOCS1^+/-^ heterozygous littermates, but soon deteriorate and decease around their third week of life. Histology revealed monocytic organ infiltration and fatty liver degeneration as a result of generalized hyperinflammation (24, 25). Additionally, smaller thymi and a progressive loss of B-lymphocyte maturation in bone marrow, spleen, and peripheral blood were observed (24). Within the T-cell compartment, a rapid reduction in thymocytes, expansion of CD4+ and CD8+ T cells in the bone marrow and an increased proliferative response to stimulation with IL-2 were measured in (SOCS1^-/-^) compared to healthy mice (26). Strikingly, early lethality in (SOCS1^-/-^) mice could be rescued in additional double RAG2 (or IFN-γ receptor) knockouts (25, 26).

Whereas heterozygous (SOCS1^+/-^) mice do not present with neonatal onset hyperinflammatory phenotypes, they accumulate autoimmune manifestations including development of anti-ssDNA and anti-dsDNA antibodies, as well as inflammatory changes in lungs, salivatory glands and kidneys. These changes predominate in female mice which is in parallel with the female gender-biased incidence in human SLE (27). These observations in murine models led to the hypothesis that SOCS1 deficiency expedites autoimmune phenotypes, and that total SOCS1 deficiency might be fatal in humans.

### 2.7 Human SOCS1-haploinsufficiency

The first reported cases of SOCS-HI date back to 2020 (28). In line with the above insights from nonprimates, one of the initially reported SOCS1-deficient patients presented with severe Multisystem Inflammatory Syndrome in children (MIS-C) after SARS-CoV-2 infection, resulting in increased type I and type II interferon signaling (29, 30). Since then, over 10 families with SOCS1-HI have been reported, presenting a broad phenotypic spectrum from autoimmune diseases, autoinflammatory manifestations to infection susceptibility and cancer (**Figure 1B**).

The most “common” phenotype is SLE-like with cutaneous, renal, and articular involvement (31–33). Other organ autoimmunity includes hepatitis, pancreatitis, thyroiditis, coeliac disease and alopecia (11, 31). Additionally, autoimmune cytopenias such as immune thrombocytopenia (ITP) or autoimmune hemolytic anemia (AIHA) are quite common (11, 29, 31, 32, 34). Another group of patients present with autoinflammatory phenotypes, as for instance, allergic diseases like asthma or rhinoconjunctivitis, and with granulomatous organ involvement such as granulomatous lymphocytic interstitial lung disease (GLILD), organizing pneumonia, and granulomatous uveitis (11, 34). Skin involvement is relatively common and may present as atopic skin disease (11) or psoriasis-like lesions (31, 35). Less frequent gastrointestinal signs include coeliac disease, oral ulcers, Crohn’s-like diseases or chronic intestinal pseudo-obstruction (CIPO) (11, 31, 36). While infections are usually localized and non-invasive (29, 35), some severe, life-threatening bacterial infections have occurred (11, 34). Additionally, lymphadenopathy, mimicking ALPS-like diseases (31, 34), and Hodgkin’s lymphoma (31) have been reported in the literature (see **Figure 1B**). While SOCS1 alterations are commonly observed in human malignant diseases, only one SOCS1-haploinsufficient (HI) patient has been reported to develop a malignancy. This may be attributed to the dichotomous effects of SOCS1 on tumorigenesis, suggesting a loss of its oncogenic effects in SOCS1-HI individuals. Larger cohort studies, i.e. of the presently increasing number of SOCS1-HI patients may shed light on this matter and unveil specific cancer occurrences. According to the International Union of Immunological Societies (IUIS), SOCS1 deficiency falls under diseases of immune dysregulation (Group IV) (37). In contrast to murine SOCS1 deficiency, so far described SOCS1-haploinsufficient (HI) patients generally do not exhibit lymphopenia or specific impairments in lymphocyte differentiation (11, 24, 29, 31). SOCS1-HI patients often demonstrate a skewing towards Th1-phenotypes and elevated levels of various pro-inflammatory cytokines in their serum (11, 29, 31). Similar to observations in humans, elevated baseline levels of IFN-γ have been particularly noted in SOCS1-deficient mice (11, 25, 26, 29, 31). Interestingly, while deficiency of T cells isolated from the thymus (thymocytes) has been observed in SOCS1-deficient mice (25), as well as dysfunction of Tregs in both homozygous (SOCS1^-/-^) and heterozygous (SOCS1^+/-^) mice (27) SOCS1-HI patients tend to have reduced levels of Tregs (11, 31).

Dysregulated innate viral defense mechanisms induce hyperactive JAK/STAT-signaling and Type-I-mediated gene expression reminiscent of type-I-interferonopathies, presenting with usually severe autoinflammatory diseases such as SLE-type or Aicardi-Goutières-syndrome (38, 39). Despite the overactivation of JAK/STAT-signaling in SOCS1-HI, we do not see upregulated Type-I Interferon mediated gene expression in all affected individuals (11). We hypothesize that infectious or other triggers may destabilize this equilibrium leading to a hyperinflammatory state.

SOCS1-HI manifests as a genetically heterogeneous disease with no straightforward structure-function relations: The location of the different variants meanwhile identified does not predict clinical manifestations or disease severity. Complete heterozygous deletions of SOCS1 (32) may exhibit less severe phenotypes than heterozygous C-terminal variants (34). As in other inborn errors of immunity with autosomal dominant inheritance, family members with SOCS1-HI may show an incomplete clinical penetrance (31). The majority of reported variants are frameshift or missense variants. A recent report highlights two “benign” variants which - while not disrupting SOCS1 function *in vitro* - contribute to an immune dysregulatory phenotype when in *cis* (33). The genetic spectrum of SOCS1-HI is diverse and underscores the complexity of this condition, as depicted in **Figures 1A, B** and in **Supplementary Table S1**.

### 2.8 Treating SOCS1-haploinsufficiency

Knowledge about molecular mechanisms of SOCS1 in intracellular cytokine signaling and discovery of SOCS1-deficiency in humans, allow for speculation on therapeutic strategies. One of the most promising and clinically accessible avenues involves the JAKinhibition (JAKi) with approved drugs like ruxolitinib, baricitinib, or tofacitinib. These molecules selectively block different JAKs thereby reducing the activity of one or more JAK-isoforms, and decreasing intracellular inflammation (40). Indications with treatment approval include hematologic disorders such as myelofibrosis, acute and chronic graft-versus-host disease (GvHD), rheumatological disorders such as rheumatoid arthritis and SLE, dermatological disorders such as atopic dermatitis, psoriasis, vitiligo and alopecia, and inflammatory bowel diseases (41).

In the initial large-cohort study of SOCS1-haploinsufficient patients, Hadjadj et al. presented compelling *in vitro* and *in vivo* data for effective inflammation control through JAKi treatment (31). In their experiments, EBV-transformed patient B cells stimulated with IFN-γ in the presence of ruxolitinib, showed reduced phosphorylation of STAT1 and STAT5, as well as decreased mRNA expression of STAT-1 regulated genes. Moreover, STAT-1 phosphorylation was reduced in patients’ monocytes treated with varying baricitinib dosages (31).

Encouraged by these findings, clinicians embraced in-label use of JAK inhibitors in SOCS1-HIi patients with manifestations of autoinflammatory diseases. In 2022, a patient with heterozygous SOCS1-deletion and severe enthesitis-related arthritis showed significant improvement upon tofacitinib treatment, even stabilizing immunocytopenia (ITP) that was poorly controlled with conventional immunosuppression (32).

Recent discoveries by Rodari et al. unveiled two novel SOCS1 variants in patients with severe gastrointestinal manifestation (36). *In vitro* data demonstrated normalized STAT1 phosphorylation, and *in vivo* treatment with ruxolitinib resulted in both clinical and laboratory improvements, showcasing reduced pro-inflammatory cytokines in the patient’s plasma.

While JAK inhibitors are shown to be clinically effective, cytokine profiles of treated patients revealed that the massive IFNγ upregulation in SOCS1-HI patients was only marginally affected by JAKi treatment (36). This raises the prospect of targeted IFNγ treatment as a potential therapeutic option for controlling SOCS1 hyperinflammation and immune dysregulation for selected patients. Emapalumab, a monoclonal antibody against IFNγ, has been approved for treating refractory, recurrent, or progressive primary HLH since 2018 (42). Exploring IFNγ as a therapeutic target for SOCS1-HI in future *in vitro* studies is a promising option especially in highly dysregulated immune conditions such as MIS-C. Prior administration of Emapalumab, it is imperative to exclude mycobacterial and viral infections particularly in hemophagocytic syndrome (HPS) patients (43). The careful consideration of benefits and potential side effects is paramount in maximizing the utility of this approach.

Whilst investigating potent anti-inflammatory drugs to regulate hyperactive JAK/STAT signaling, attention has turned towards studying SOCS-mimetic peptides. These molecules are small peptide sequences that are to mimic the protein function. They are coupled to a delivery sequence to penetrate cells (44, 45). The identification of fatal consequences stemming from the lack of SOCS1 across a spectrum of autoimmune, autoinflammatory, and malignant diseases has prompted the exploration of SOCS1 mimetic peptides in various animal experiments especially in the condition of recurrent uveitis (44). SOCS1-mimetic peptides may also be produced in such a way that only distinct parts of the protein such as the KIR are mimicked, which could be helpful in SOCS1-HI variants with residual protein activity. The use of SOCS1 mimetic peptides offers a potential avenue for controlling inflammation. In future pharmacological studies, challenges like high costs, low permeability, difficulties of intracellular delivery, proteolytic instability, lack of targeted delivery and poor oral bioavailability need to be addressed (44). SOCS1 mimetic peptides have also been suggested in the context of SARS-CoV-2 and other viral infections (46). To date, only experimental therapeutic approaches have been tested, the successful application in humans is still to be investigated (47).

While SOCS1’s role extends beyond JAK/STAT signaling, with involvement in ubiquitin ligase formation, future studies may explore its potential as a target for disease control by regulating proteasomal degradation.

**Figure 2:**
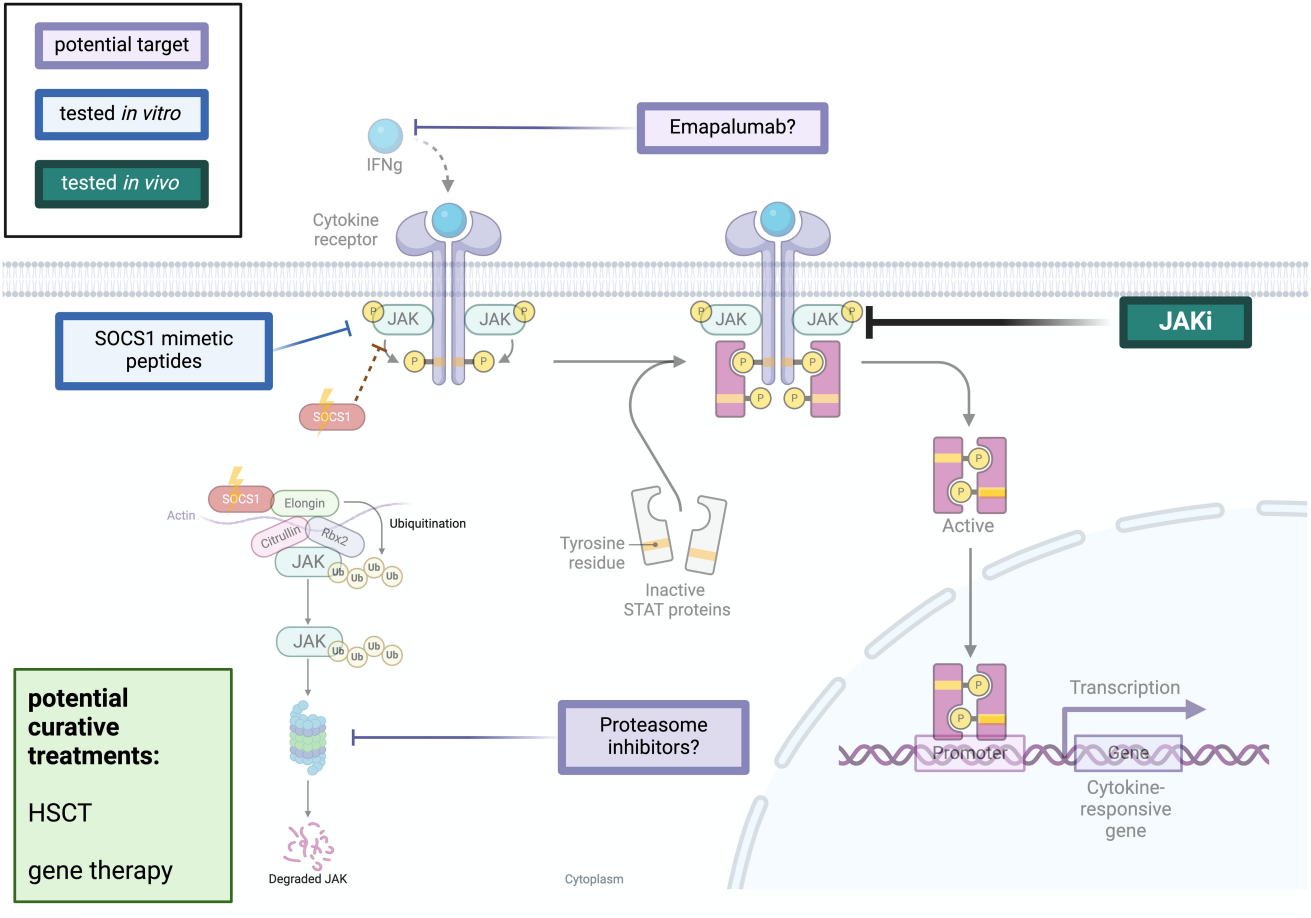
Potential treatment options for SOCS1-haploinsufficiency JAK/STAT-intracellular signaling and proteasomal degradation as well as potential therapeutic options for SOCS1-HI patients. The green rectangle lists potential curative treatment options for SOCS1-HI which have not been applied in humans so far. The turquoise rectangle shows JAKi treatment which has been used successfully in human SOCS1-HI. The blue rectangle lists SOCS1-mimetic peptides which are only used in experimental settings and not in human studies. Violet rectangles name potential therapeutics administered in other human diseases with different mechanisms of action the might potentially beneficial in treatment of SOCS1-HI on a mechanistical basis. The green rectangle in the lower left part of the illustration offers further perspective curative treatments which have not been performed in humans so far. The figure was created using biorender.com.

As IEI with severe immune dysregulation and potentially life-threatening infections and malignant diseases, severely affected SOCS1 patients might be eligible for HSCT. As in other (adult) IEI patients, optimal donor selection and clinical state before HSCT are important criteria accompanying this decision (48). Furthermore, it remains unclear if the correction of the hematopoietic compartment will result in complete cure as SOCS1 is also expressed in other tissues (11). To date, there are no SOCS1-HI patients reported that underwent HSCT as curative treatment (summarized in **Figure 2**).

## 3 Discussion and concluding remarks

The recent discovery of SOCS1-HI as a human inborn error of immunity (IEI) has profoundly transformed the landscape of SOCS1 research. Initially believed to be incompatible with human life, the discovery of SOCS1-HI has challenged the research community. Over the past three years, an increasing number of reported SOCS1 variants and clinical case studies suggest that SOCS1-HI might be an underdiagnosed IEI. This trend is further underscored by the establishment of various SOCS1 study groups worldwide (European SOCS1 study group from the European society for Immunodeficiencies (ESID) registry and ESID-registry associated SOCS1 sub registry (49), US SOCS1 study group (34) that aim to review patient data scientifically, signifying the global interest and collaborative efforts in unraveling the complexities of this condition.

To appreciate the disease spectrum, it is crucial to evaluate novel genetic SOCS1 variants for their functional consequences *in vitro* as well as *ex vivo* using patients’ primary cells. This approach allows for a nuanced understanding of the clinical phenotypes. Currently it remains unclear why SOCS-HI occurs with very different disease severities requiring individualized therapeutic approaches, as is known for other rare IEIs (50). At present, a review of the published data on clinical, immunological and genetic data on SOCS1-HI suggests a lack of a straightforward structure-function relationship: intriguingly, complete SOCS1 deletions may be associated with milder phenotypes compared to C-terminal variants. Furthermore, within SOCS1-HI families, members who carry one variant exhibit no clinical symptoms, while relatives with the exact same SOCS1-variant manifest a severe phenotype. Unraveling the mechanisms responsible the clinical penetrance of this disease will be a focal point of future studies. In order to assure the pathogenicity of a given SOCS1 variant, studying pathways in which SOCS1 is involved, is perspectively key to guide clinicians and patients in deciding whom to treat and when to start which treatment. The future of managing SOCS1-HI is to involve individualized treatment approaches, incorporating *in vitro* functional drug evaluation as part of point-of-care interventions.

Focusing on curative treatments, the evaluation of hematopoietic stem cell transplantation may be a potential option for patients with SOCS1-HI. Additionally, gene therapy emerges as a promising therapeutic approach that warrants further exploration as a curative treatment option. As research progresses, these efforts promise to unveil novel insights into SOCS1-HI and pave the way for more effective, personalized treatments.

## Data availability statement

The original contributions presented in the study are included in the article/**Supplementary Material**. Further inquiries can be directed to the corresponding author.

## Author contributions

JK: Writing – review & editing, Writing – original draft, Visualization, Investigation, Conceptualization. LC: Writing – review & editing, Writing – original draft, Supervision, Investigation. TS: Writing – review & editing, Writing – original draft. CS: Writing – review & editing, Writing – original draft, Supervision. AD: Writing – review & editing, Writing – original draft, Supervision, Conceptualization.
