# Supplementary material for: One gene to rule them all – clinical perspectives of a potent suppressor of cytokine signaling – SOCS1
Source: Front Immunol. 2024 Apr 22;15:1385190. doi: 10.3389/fimmu.2024.1385190 (PMC11070515; doi:10.3389/fimmu.2024.1385190)
Supplement: Supplementary file 1 [file Table_1.docx]

| **publication** | **patient description** | **SOCS1 variant** | **sex** | **age at symptom onset (years)** | **autoimmunity** | **autoinflammation** | **lymphoproliferation/**  **malignancy** | **infections** | **other phenotypes** | **therapy** |
| --- | --- | --- | --- | --- | --- | --- | --- | --- | --- | --- |
| Hadjadj et al. 2020 | A1 | c.368C>G  p.P123R | female | 2 | ITP |  |  |  |  | Corticosteroids, IVIg, TPOr agonist, MMF |
|  | A2 | c.368C>G  p.P123R | female | 6 | ITP, thyroiditis | polyarthritis |  |  |  | Corticosteroids, hormonal substitution |
|  | B1 | c.24delA  p.A9Pfs*76 | female | 5 | Evans syndrome |  | adenopathy, splenomegaly | bronchopulmonary |  | Corticosteroids, IVIg, Rapamycine |
|  | B2 | c.24delA  p.A9Pfs*76 | male | 3 |  | coeliac disease, psoriasis | Hodgkin lymphoma |  |  | Topical treatment |
|  | C1 | c.476_480dupGCCGC  p.M161MAfs*46 | female | 3 | Evans syndrome |  | adenopathy, HSM |  |  | Corticosteroids, MMF |
|  | D1 | c.64C>T  p.R22W | male | 16 | SLE |  |  |  |  | Corticosteroids, Hydroxychloroquine, MMF |
|  | E1 | c.460T>C  p.Y154H | female | 9 | SLE |  |  |  |  | Corticosteroids, Hydroxychloroquine, Methotrexate, Cyclophosphamide, MMF, Baricitinib |
|  | E2 | c.460T>C  p.Y154H | female | 16 | ITP |  |  |  |  | Corticosteroids, IVIg, Hydroxychloroquine, Azathioprin, Rituximab, Splenectomy |
|  | E4 | c.460T>C  p.Y154H | male | 15 |  | psoriasis |  |  |  | Topical treatment |
|  | E5 | c.460T>C  p.Y154H | female | 44 | autoimmune hepatitis | psoriasis, spondyloarthritis |  |  |  | Corticosteroids, Hydroxychloroquine, Methotrexate, Anti-TNFα therapy |
| Lee et al. 2020 | P1 | c.108delG  p.Ala37Argfs∗48 | male | 5 months | AIN, ITP, AIHA |  |  | Otitis media |  | Corticosteroids, MMF |
|  | P2 | c.24delA  p.Ala9Profs∗76 | male | 14 | AIN, ITP, AIHA | MIS-C |  |  |  | Corticosteroids, MMF, IVIG, Eltrombopag |
| Koerholz et al. 2021 and Thaventhiran et al. 2020 | P1 | c.(192C>G)  p.(Tyr64*) | female | 5 | Neutropenia, AIHA, ITP, Alopecia  totalis | Atopic eczema,  allergic rhino-  conjunctivitis, allergic  asthma | HSM | Pneumonia with  URI empyema, dental abscesses, UTI, local HSV  infections, shingles |  | Prednisolone  IVIG  Romiplostim |
|  | P2 | c.(192C>G)  p.(Tyr64*) | male | 7 | Hashimoto thyroiditis | allergic rhino-  conjunctivitis, allergic  asthma, pernicious anemia, EAA | splenomegaly | URI |  | Prednisolone  Levothyroxine, Vitamin  B12 |
|  | P3 | c.(480_481insGCGGC)  p.(Met161Alafs*46) | female | 8 | ITP, AI-Hepatitis | chronic granulomatous uveitis, GLILD | splenomegaly | URI, Pneumonia Otitis media |  | Prednisolone,  Rituximab  IVIG |
| **publication** | **patient description** | **SOCS1 variant** | **sex** | **age at symptom onset (years)** | **autoimmunity** | **autoinflammation** | **lymphoproliferation/**  **malignancy** | **infections** | **other phenotypes** | **therapy** |
| Michniacki et al. 2022 | P1 | 5 MB microdeletion at 16p13.2p13 | female | 5 | bone marrow hypoplasia, ITP | MIS-C, enthesitis, arthritis |  |  |  | tofacitinib |
| Du et al. 2023 | P1 | c.149 C > T and c.227C > G (in cis)  p.Pro50Leu and p.Ala76Gly | female | 13 | SLE | peritonitis |  |  | posterior reversible encephalopathy syndrome | pulse-dose glucocorticoids, IVIG, plasmapheresis, cyclophosphamide, rituximab, MMF, hydroxy- chloroquine |
|  | P2 | c.149 C > T and c.227C > G (in cis)  p.Pro50Leu and p.Ala76Gly | male | 10 | ITP |  |  |  |  | Prednisone, IVIG, hydroxychloroquine |
| Hale et al. 2023 | P1 | c.480_481insGCGGC  p.Met161Alafs*46 | female | 7 | ITP, AIN |  | HSM, lymphadenopathy | Severe pneumonia, fungemia | non-cirrhotic portal hypertension | IVIG, G-CSF, sirolimus, liver/lung transplant |
|  | P2 | c.480_481insGCGGC  p.Met161Alafs*46 | male | 5 | hepatitis | recurrent fever, eczema, allergic rhinitis | HSM, lymphadenopathy |  |  |  |
| Rodari et al. 2023 | P1 | c.58C>T  p.Arg20* | female | 18 |  | Psoriasis, Crohn’s disease |  | Severe chronic rhinosinusitis |  | steroid, azathioprine, infliximab, adalimumab, ustekinumab |
|  | P2 | c.298_301dup  p.Phe101Tyrfs*17 | female | 24 |  | anosmia, Asthma bronchiale, chronic intestinal pseudo-obstruction, leiomyosistis, bipolar aphtosis, Raynaud’s syndrome, severe arthritis | Meningioma |  |  | Systemic steroid, Bude- sonide, ruxolitinib |
| Gruber et al. 2023 | P1 | c.202_203delAC  p.Thr68fs | male | 10 |  | dermatitis, polyarthritis, enthesitis, fever, psoriasis |  | MSSA+ skin abscesses, pneumonia |  | Secukinumab, dupilumab |

supplementary table S1 - genotype-phenotype correlation and therapeutic options of all cases of SOCS1 haploinsufficiency reported to date. ITP: immune thrombocytopenia; SLE: systemic lupus erythematosus; HSM: hepatosplenomegaly; IVIG: intravenous immunoglobulins; TPOr: thrombopoietin receptor; MMF: Mycophenolat Mofetil; TNFα: tumor necrosis factor α; AIN: autoimmune neutropenia; AIHA: autoimmune hemolytic anemia; URI: upper respiratory infection; UTI: urinary tract infection; HSV: herpes simplex virus; MIS-C: Multisystem Inflammatory Syndrome in Children; EAA: exogenous allergic alveolitis; GLILD: granulomatous-lymphocytic interstitial lung disease; G-CSF: granulocytes-colony-stimulating factor; MSSA: methicillin-susceptible S. aureus
